# Effect of environmentally-relevant concentrations of nonylphenol on sexual differentiation in zebrafish: a multi-generational study

**DOI:** 10.1038/srep42907

**Published:** 2017-02-23

**Authors:** Dong Sun, Qi Chen, Ning He, Pan-pan Diao, Li-xing Jia, Shun-shan Duan

**Affiliations:** 1 Research Center of Hydrobiology, Key Laboratory of Aquatic Eutrophication and Control of Harmful Algal Blooms of Guangdong Higher Education Institute, Jinan University, Guangzhou 510632, China

## Abstract

Nonylphenol (NP) is a persistent environmental chemical that can disrupt the organism’s endocrine system, and is detected in the surface water and sea. In this study, we investigated whether NP can alter transcriptional expression of sexual differentiation-related genes. Three generations of zebrafish were exposed to 0, 2, 20 and 200 μg·L^−1^ of NP, and transcriptional expression of sexual differentiation genes were assessed in 10, 20 and 40 dpf in the F1 and F2 generations. Growth of zebrafish exposed to 200 μg·L^−1^ of NP was inhibited at 125 dpf in the F1 generation. 20 μg·L^−1^ of NP resulted in 80% females in the F1 generation, but had no effect on the F2 generation. In terms of the sexual differentiation genes, the transcriptional expression of cyp19a1a and esr1 genes were upregulated in 20 μg·L^−1^ of NP in the F1 generation. But expression of the sexual differentiation genes were not affected in the F2 generation. Overall, NP could affect sexual differentiation and gene transcriptional expression in the F1 generation. The tolerance of contaminant in the offsprings was improved at low concentration.

Nonylphenol (NP) is a well-known endocrine disrupting chemical (EDC) that is persistently and frequently found in rivers, tap water, lakes and sea^1^,^2^. NP, arising from the degradation of NP ethoxylates, is a highly cost-effective surfactant used in industrial, commercial and household applications^3^,^4^. NP is a widely distributed in aquatic system. The concentration of NP ranges from undetected to 33.2 μg·L^−1^ in lakes and rivers^5^, 330 μg·L^−1^ in sewage effluent and 180 μg·L^−1^ in downstream wastewater treatment facilities^6^. Meanwhile, Li *et al*. found that NP in human urine samples ranged from 6.95 μg·L^−1^ to 29.38 μg·L^−1^ in Guangzhou^7^. Hence, it is necessary to assess interferential responses of environmentally-relevant concentrations of NP on aquatic organisms.

NP is exogenous estrogen, which can bind to estrogenic receptors like endogenous estrogen mimics^8^. In addition, it may accumulate in organisms in sufficient concentrations to induce similar effects as Estradiol (E2)^9^. NP exerts numerous direct and indirect endocrine disruption effects, which interfere with normal physiological and developmental signaling in different species^10^,^11^. Legler *et al*. showed human exposed to EDCs could cause intelligence quotient (IQ) loss, intellectual disability, autism, etc^12^,^13^,^14^. Thus, NP may change the genome and epigenome resulting in disease^15^, and transfer these adverse health effects to the next generation^16^,^17^. NP was reported to have toxic effects on zebrafish^18^, rainbow trout^19^, Japanese medaka^20^, fathead minows^21^, Atlantic salmon smolts^22^, Nile tilapia^23^,^24^, etc. But effects of environmentally-relevant concentrations of NP on future generations is still lacking.

The estrogenic effect of NP interfered with sexual differentiation in teleosts. Norrgren *et al*.^25^ showed that high, but not low, concentrations of NP affected sexual differentiation in Atlantic salmon. In contrast, Hill and Janz^26^ found that the number of females in 100 μg·L^−1^ groups of NP was significantly higher than the control at 60 days post fertilization (dpf), whereas the sex ratio was similar to control at 120 dpf of NP in zebrafish. However, Ackermann *et al*.^27^ found that 10 μg·L^−1^ of NP in sewage treatment effluents and some rivers did not affect sexual differentiation of wild rainbow trout. These data indicated that NP can affect the teleosts at environmentally-relevant concentrations.

Studies on the effects of environmental hormones on multi-generations of organisms have gradually increased in recent years. Previous studies showed that sex ratio was an effective biomarker for the assessment of EDCs^28^. Coimbra *et al*.^29^ found that exposure to clofibric acid improved the male ratio in the F1 generation, but had no effect on the F2 generation. Corrales *et al*.^30^ reported similar results and showed that mortality significantly increased in higher dose groups of (Benzo A Pyrene) Bap (2.3 and 20 μg·g^−1^) in the F1 generation, with no differences in the F2, F3 and F4 generations in zebrafish larvae. It was suggested that the parental effects were transmitted to the offsprings, which altered their DNA sequences and enhanced their ability to tolerate stressful conditions^31^. Therefore, sex ratio as an environmental marker to assess EDCs needs further study. In fact, multigenerational exposure of aquatic life has unknown effects at the population level and the expression of sexual differentiation genes at each generation.

The objectives of the present study were: 1. to determine the effect of NP on sexual differentiation in zebrafish. For this purpose, all life stages of the fish were continuously exposed to 2, 20 and 200 μg·L^−1^ of NP for three generations (P, 30 dpf; F1, 125 dpf; F2, 140 dpf), 2. To determine the expression pattern of sexual differentiation genes. For this purpose, the effect of NP exposure on sexual differentiation at the transcriptional level was examined in two generations (F1 and F2) at 10 dpf, 20 dpf and 40 dpf concentrations, respectively.

## Results

### Measured concentration of NP

The measured concentrations of NP at the beginning of exposure and water renewal (24 h and 48 h) are shown in Table 1. The measured concentrations were close to the nominal concentrations. Meanwhile, NP was not detected in the control and solvent control groups.

### Length of zebrafish at NP exposure

**Figure 1:**
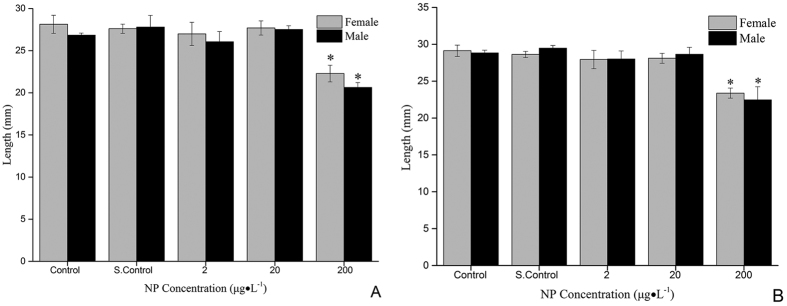
Growth of zebrafish exposed to 2, 20 and 200 μg·L^−1^ NP. (**A**) F1 generation exposed to NP at 125 dpf. (**B**) F2 generation exposed to NP at 140 dpf. S. Control is solvent control. Significant difference between the exposure group and the control. *p < 0.05.

Length was determined in the F1 generation at 125 dpf and in the F2 generation at 140 dpf. In the F1 generation (Fig. 1), a significant decrease (p < 0.05) in the length was observed in fish exposed to 200 μg·L^−1^ of NP at 125 dpf. Also, a significant decrease (p < 0.05) in the length was observed at 140 dpf in the F2 generation.

### Sex ratio in the multi-generation exposed to NP

**Figure 2:**
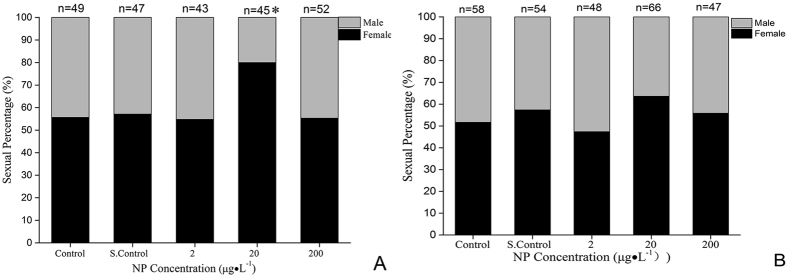
Sex ratios of zebrafish exposed to 2, 20 and 200 μg·L^−1^ NP. Data are expressed as the percentage of males and females. (**A**) F1 generation exposed to 125 dpf of NP. (**B**) F2 generation exposed to 140 dpf of NP. S. Control is solvent control. Significant difference in sex ratio from control: *p < 0.05. (Supplement Figures from 3-A to 3-F)

The gender (Fig. 2) was determined in the F1 generation at 125 dpf and F2 generation at 140 dpf. In the F1 generation, the proportion of females accounted for 80% (36 females) in 20 μg·L^−1^ of NP. Treatment with 2 μg·L^−1^and 200 μg·L^−1^ did not show any significant difference from the control. In the F2 generation, 20 μg·L^−1^ had a significant recovery than the F1 generation, but the proportion of females was still higher than for other treatments.

### Gene transcriptional expression after exposure to NP

**Figure 3:**
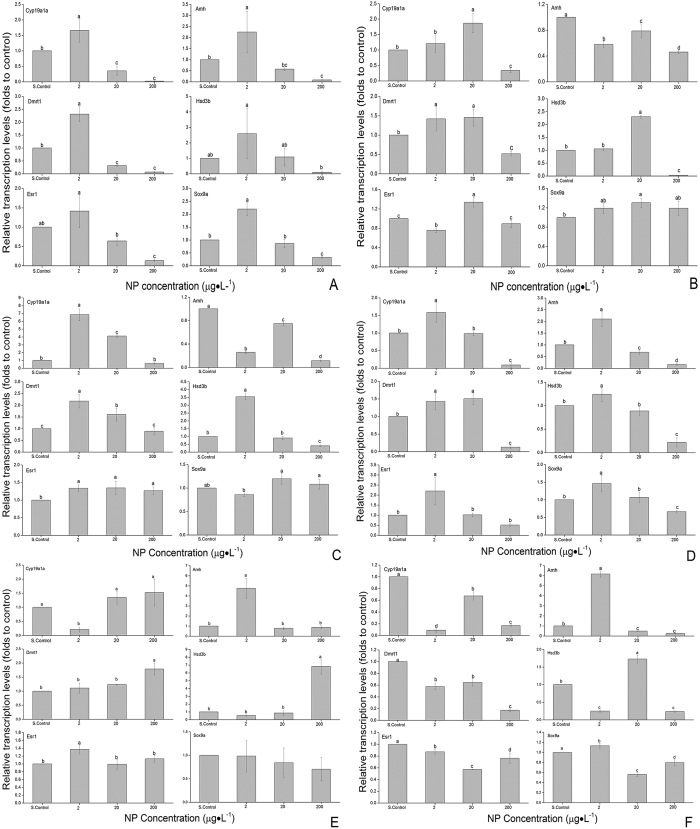
Effect of S. Control, 2, 20 and 200 μg·L^−1^ of NP on sexual differentiation genes in zebrafish exposed for two generations. (**A**) F1 generation exposed at 10 dpf to NP. (**B**) F1 generation exposed at 20 dpf to NP. (**C**) F1 generation exposed at 40 dpf to NP. (**D**) F2 generation exposed at 10 dpf to NP. (**E**) F2 generation exposed at 20 dpf to NP. (**F**) F2 generation exposed at 40 dpf to NP. S. Control is solvent control. Different letters indicate significant differences at p = 0.05.

Figure 3A shows that after exposure at 10 dpf, cyp19a1a, amh, dmrt1, hsd3b, esr1 and sox9a expression levels were significantly higher in the 2 μg·L^−1^ group than in the other treatment groups, lower in the 200 μg·L^−1^ group, and unaffected in the 20 μg·L^−1^ group. Figure 3B shows that after exposure at 20 dpf, the expression levels of these genes in the 20 μg·L^−1^ group were significantly higher than in the other treatment groups, and significantly lower in the 200 μg·L^−1^ group than in the other groups. While at 40 dpf, the expression levels of cyp19a1a, dmrt1, esr1 and hsd3b were significantly higher in the 2 and 20 μg·L^−1^ groups than in the solvent control (Fig. 3C).

Figure 3D shows that expression of these genes in F1 at 10 dpf, in 2 μg·L^−1^ were significantly higher than in solvent control, and in 200 μg·L^−1^ were lower than in solvent control. In F2 at 20 dpf (Fig. 3E), expression of cyp19a1a in 2 μg·L^−1^ was significantly lower, while gene expression was not affected in 20 and 200 μg·L^−1^ as compared to the solvent control. In F2 at 40 dpf (Fig. 3F), cyp19a1a and esr1 expression levels were significantly lower in all treated groups than in solvent control, while amh was higher in 2 μg·L^−1^ than in other treatments.

## Discussion

This study showed that NP can behave as a strong estrogen agonist at environmentally-relevant concentrations in zebrafish. The estrogen effect of NP potentially disrupted the growth and sexual differentiation of zebrafish. Full life cycles of two generations showed that NP at 2 and 20 μg·L^−1^ concentrations did not affect morphological and toxicological endpoints in zebrafish, and 200 μg·L^−1^ concentration inhibited growth of zebrafish. This indicated that 200 μg·L^−1^ of NP was acutely and chronically toxic to zebrafish, not only for its estrogenic effect. Lin and Janz^32^ reported that 10 and 100 μg·L^−1^ of NP had no effect on growth of zebrafish. However, another study showed that 100 μg·L^−1^ of NP inhibited growth in terms of relative body length of rainbow trout^33^. Thus, different results were observed in different species.

We found 80% females in the 20 μg·L^−1^ NP group at the F1 generation, with no effect on the sex ratio of zebrafish in the 2 and 200 μg·L^−1^ NP groups. The sex ratio was similar to the control in the F2 generation. Another study had shown similar results whereby 100 μg·L^−1^ of NP exposure resulted in 100% females at 60 dph. Meanwhile, 10 μg·L^−1^ of NP exposure resulted in 57.9% females, which was higher than in the control group. However, 100 μg·L^−1^ of NP resulted in 75.8% males at 120 dph^32^. Also Hill *et al*.^26^ reported similar results that females were significantly higher after 100 μg·L^−1^ of NP exposure than in the control at 60 dph, while 30 μg·L^−1^ group had the most females at 160 dph. The results of the 20 μg·L^−1^ group in the F1 generation of the present study were in accordance with those previously reported, but the 20 μg·L^−1^ group in the F2 generation was inconsistent with previous studies. 200 μg·L^−1^ of NP inhibited the growth in F1 and F2 generations, which indicated that NP’s effect on growth was worse than endocrine disruption. Ackermann^27^ reported that 10 μg·L^−1^ of NP had no effect on the sexual differentiation in rainbow trout, which could be due to the difference in species. Parental exposure to contaminants can significantly affect offsprings’ characteristics and improve their fitness^34^. The observed differences in sex ratios between the F1 and F2 generations may be related to contaminant tolerance of offsprings from parental exposure.

The sex determination mechanisms of zebrafish differ from other teleosts. Zebrafish has polygenic sex determination and unknown sex determination genes^35^. The sox9, dmrt1, hsd3b and amh genes are thought to regulate gonad sex differentiation^36^. The cyp19a1a and esr1 genes are related to aromatase, which convert androgens to estrogen and control the balance of sex steroids^37^. The amh, dmrt1, hsd3b and sox9a genes were related to male cells development^38^,^39^. In this present study, NP had strong estrogen effects on sex differentiation, leading to the change of sex ratio in the F1 generation. The transcriptional expression of genes in sex determination and differentiation were altered. A statistically significant upregulation of cyp19a1a and esr1 was seen in the 20 μg·L^−1^ NP group at 20 dpf in the F1 generation. These results were consistent with the shift of sex ratio towards females in the F1 generation. Nevertheless, amh, dmrt1 and hsd3b genes were also significantly upregulated, but had no effect on the sex ratio. The cyp19a1a and esr1 genes had no effect on the sex ratio in the 20 μg·L^−1^ NP group at 20 dpf in the F2 generation. The sex ratio of the F2 generation was the same as the control. Alterations of cyp19a1a and esr1 genes may contribute to female differentiation after exposure to 20 μg·L^−1^ of NP at 20 dpf.

The present study showed that NP could cause sex differentiation at environmentally-relevant concentrations. Changes in the transcriptional expression of cyp19a1a and esr1 genes may decide the sex ratio of zebrafish after exposure to NP. However, the shift in sex ratio towards females after exposure to NP did not occur in each generation. With the extension of exposure time and metagenesis, the offspring contaminant tolerance was improved. These results implied that NP could cause potential effects on sex differentiation by altering transcriptional expression of genes in zebrafish.

## Materials and Methods

### Chemicals

NP (99% purity) was purchased from Aladdin, dissolved in methanol to obtain a 1 g·L^−1^ stock solution and stored at −20 °C in the dark.

### Fish husbandry and reproduction

Four hundred of three-month-old zebrafish (*Danio rerio*) were purchased from a local supplier. They were transferred to the laboratory and acclimated for at least one month in flow-through holding tanks supplied with aerated freshwater, kept at 26 ± 1 °C with a photoperiod of 14 h:10 h light: dark. The fish were fed three times a day, with Artemia naupii at noon, and blood worm at morning and night.

Embryos were obtained from adults which placed in tanks in groups of two males and one female for each concentration (Control, Solvent Control, 2, 20 and 200 μg·L^−1^) overnight. These fishes were placed with partitions in a specific aquarium. Mesh bottoms in the aquarium protected the embryos from being eaten. The partition was removed next morning, and spawning was stimulated by the onset of light. Embryos were collected from each tank after one hour, and examined under a microscope at 6 hpf (hours post-fertilization). The normally developed embryos were selected for subsequent experiments. All animal protocols were approved and performed in accordance with Animal Experiment Committee of Research Center of Hydrobiology in Jinan University.

### Measurement of NP in exposure solutions

To measure the actual concentrations of the exposure, media were collected from each tank twice in one month. The exposure concentrations of NP were extracted with the previous methods^40^. Briefly, NP concentration was detected in 20 mL of collected water samples using solid phase extraction (SPE). The water samples were filtered through glass fiber filters (Whatman GF/F, 0.7 μm effective pore size, UK). The solid phase extraction element was conditioned using 10 mL of methanol followed by 10 mL of redistilled water. The filtered water was passed through the SPE cartridges at 5–10 mL/min. After one hour, the target compounds were eluted from the cartridges using 7 mL methanol and 5 mL dichloromethane. The extracts were dried under a gentle nitrogen stream, and then dissolved in 1 mL methanol in a glass vial, triplicate. These vials were kept at −18 °C. Accurate concentrations of 1, 2, 10, 50, 100, 200 μg·L^−1^ of NP were used as the external standard to make the standard curve for calculating the actual concentration. All samples were analyzed by liquid chromatography-tandem mass spectrometry (LC-MS/MS).

### Exposure assays

**Figure 4:**
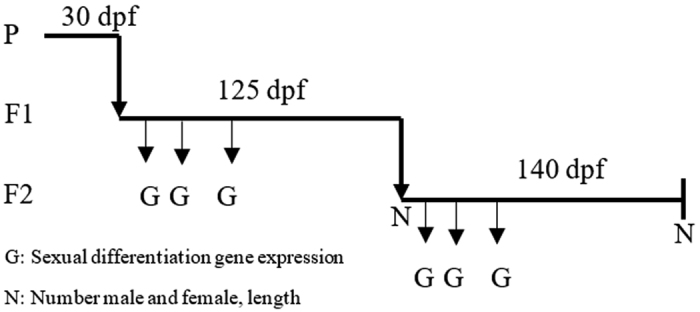
Schematic representation of exposure period, time points and endpoint determined in each generation.

In order to study the effects of NP on sexual differentiation in zebrafish, a multi-generational study was performed as shown in Fig. 4.

The P generation zebrafish (acclimated for one month) were exposed to different concentrations of NP for 30 days. Thirty fishes selected randomly from the acclimated group in each tank, triplicate. After 30 days, the embryos were collected and randomly allocated according to their parental group. Exposure of the F1 generation zebrafish to the test chemicals was started at fertilization and ended at 125 dpf (days post-fertilization). All the healthy embryos were randomly kept in 15 L glass tanks containing 5 L of filtered and aerated tap water until 60 dpf, and then in 15 L glass tanks containing 10 L aerated tap water with flow through system until 125 dpf. The F2 generation embryos according to parental group were maintained for 140 dpf under the same conditions as the F1 generation. The zebrafish were exposed to NP at nominal concentrations of 0, 2, 20 and 200 μg·L^−1^ in three replicates. Methanol (0.002%) was used as the solvent for NP, so the solvent control and exposure groups received 0.002% (v/v) methanol. Half of the treatment water of each tank was renewed daily. Dead fish in all the treatment tanks were removed and recorded daily. Three fish per replicate were homogenized and stored at −80 °C for subsequent RNA extraction in 10, 20 and 40 dpf, respectively. Meanwhile, the other three fish per replicate were also stored at −80 °C as backup. The final sex ratio was determined after zebrafish reached 125 dpf and 140 dpf by checking the phenotype^41^, after anesthetization and dissection.

### Quantitative real-time PCR analysis

Total RNA was extracted from three fish from each tank using Trizol reagent (Invitrogen), as per the manufacturer’s guidelines. The quality of total RNA was determined by electrophoresis on an agarose gel stained with GoldView. RNA concentration was determined at 260 nm by Q5000 UV-Vis spectrophotometer (Quawell, USA). RNA samples with purity between 1.81 and 2.05 for ratios 260/280 were used.

1 μg of total RNA was reverse-transcribed using the cDNA synthesis kit (GoScript Reverse Transcription System, Promega, Madison, USA), as per the manufacturer’s instructions. Briefly, the total volume of 20 μL (2 μL RNA + 14 μL RNase-free water + 2 μL random primers + 2 μL Oligo(dT)_15_ primer) was incubated at 70 °C for 5 min to melt secondary structures within the template. A total volume of 20 μL of master mix solution containing 8 μL GoScript^TM^ 5X Reaction Buffer, 4 μL MgCl_2_, 2 μL PCR Nucleotide Mix, 0.8 μL Recombinant RNasin^®^ Ribonuclease Inhibition, 2 μL GoScript^TM^ reverse transcriptase and 3.2 μL RNase-free water were added to each sample. The total 40 μL reaction mixture was incubated at 25 °C for 5 min, 42 °C for 60 min and 70 °C for 15 min to stop the reaction. The cDNA was stored at −20 °C.

The qRT-PCR analysis was performed on the CFX96 Real-time System (C1000 Touch, Bio-Rad) using the GoTaq^®^ qPCR Master mix (Promega, USA), as per the instructions provided by Promega. The total volume of 20 μL contained 2 μL of cDNA sample, 7.2 μL of nuclease-free water, 0.4 μL of each primer (10 μM), and 10 μL of GoTaq^®^ qPCR Mater mix (2X). The specific primers of target and housekeeping genes are listed in Table 2. Most primer sequences were previously reported^42^. β-actin and Esr1 were constructed using primer premier 5.0 program. The melting temperature (Tm) of all primers was maintained close to 60 °C. All primers were synthesized by BGI (Guang zhou, China). The qPCR reactions were initially denatured at 95 °C for 10 min, followed by 40 cycles at 95 °C for 15 sec and 60 °C for 1 min. Melting curve analysis from 60 °C to 90 °C was performed to ensure the specificity of each amplicon. The 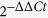 method was previously described^43^ using β-actin as a housekeeping gene to evaluate the relative transcript levels. The expression of each gene was calculated using the control group transcript levels as 1 unit. The formula is as follows: 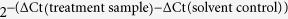


### Data and statistical analysis

All graphical illustrations and statistical analyses were made using either Origin 2015 (OriginLab Corporation, Northampton, MA, USA) and/or SPSS 19.0 software (SPSS, Chicago, IL, USA). Differences between the control and each exposure group were evaluated by one-way analysis of variance (ANOVA), followed by LSD test. A p value of less than 0.05 was considered to be statistically significant.

## Additional Information

**How to cite this article:** Sun, D. *et al*. Effect of environmentally-relevant concentrations of nonylphenol on sexual differentiation in zebrafish: a multi-generational study. *Sci. Rep.* **7**, 42907; doi: 10.1038/srep42907 (2017).

**Publisher's note:** Springer Nature remains neutral with regard to jurisdictional claims in published maps and institutional affiliations.

## Figures and Tables

**Table 1:** The exposure concentrations of NP to zebrafish in water.

Compound	Exposure time (h)	Nominal concentration (μg·L^−1^)			
		0	2	20	200
NP (NP)	0	N.D.	2.4 (0.20)	17.5 (0.38)	185.2 (0.97)
	24	N.D.	3.2 (0.27)	22.5 (0.65)	179 (1.48)
	48	N.D.	3.1 (0.35)	20.8 (0.47)	183.6 (1.59)
	average	N.D.	2.9 (0.27)	20.27 (0.5)	182.6 (1.35)

Note: Forty day old zebrafish were exposed for 30 days and the concentration of NP in the fish water was determined at 24 and 48 hours. Data are given as mean (S.D.) (n = 2 replicates).

**Table 2:** Primers for quantitative real-time PCR in zebrafish.

Gene	Sense primer (5′-3′)	Antisense primer (5′-3′)	GenBank number	Product size (bp)	reference
β-actin	CATGGCTTCTGCTCTGTATG	GCAAAGTGGTAAACGCTTCT	AF057040.1	143	
Cyp19a1a	CGGGACTGCCAGCAACTACT	TGAAGCCCTGGACCTGTGAG	NM_131154.2	264	42
Amh	TTCCTCCACGCCGACTGTAT	CCTGCCTCCTGCTGTTTGAC	NM_001007779.1	150	42
Dmrt1	TTTACCAGCCCACTCCATACTC	AGGCGGCCATTTCCACTAG	AF439562.1	85	42
Hsd3b	AGCCCATTCTGCCCATCTT	TGCCTCCTCCCAGTCATACC	AY279108.1	200	42
Esr1	TGAGCAACAAAGGAATGGAG	GTGGGTGTAGATGGAGGGTTT	NM_152959	161	
Sox9a	GCCAGGCAAAGCGGATCT	GCGGGAGGTATTGGTCAAACT	NM_131643.1	155	42
